# Microwave Absorption Properties of Graphite Nanosheet/Carbon Nanofiber Hybrids Prepared by Intercalation Chemical Vapor Deposition

**DOI:** 10.3390/nano15050406

**Published:** 2025-03-06

**Authors:** Yifan Guo, Junhua Su, Qingfeng Guo, Ling Long, Jinlong Xie, Ying Li

**Affiliations:** 1 School of Aeronautical Equipment Manufacturing Industry, Chengdu Aeronautic Polytechnic, Chengdu 610100, China; guoqingfeng629@foxmail.com (Q.G.); sclongling@163.com (L.L.); 2 Key Laboratory of Optoelectronic Technology & Systems (Ministry of Education), Center for Intelligent Sensing Technology, College of Optoelectronic Engineering, Chongqing University, Chongqing 400044, China; 20230801051g@stu.cqu.edu.cn; 3 School of Electronic Science and Engineering, University of Electronic Science and Technology of China, Chengdu 611731, China; 202111022814@std.uestc.edu.cn; 4 School of Mechanical Engineering, Chengdu University, Chengdu 610106, China; liying@cdu.edu.cn

**Keywords:** graphite nanosheet, carbon nanofiber, intercalation, chemical vapor deposition, microwave absorption

## Abstract

Carbon-based microwave absorption materials have garnered widespread attention as lightweight and efficient wave absorbers, emerging as a prominent focus in the field of functional materials research. In this work, FeNi_3_ nanoparticles, synthesized in situ within graphite interlayers, were employed as catalysts to grow carbon nanofibers in situ via intercalation chemical vapor deposition (CVD). We discovered that amorphous carbon nanofibers (CNFs) can exfoliate and separate highly conductive graphite nanosheets (GNS) from the interlayers. Meanwhile, the carbon nanofibers eventually intertwine and encapsulate the graphite nanosheets, forming porous hybrids. This process induces significant changes in the electrical conductivity and electromagnetic parameters of the resulting GNS/CNF hybrids, enhancing the impedance matching between the hybrids and free space. Although this process slightly reduces the microwave loss capability of the hybrids, the balance between these effects significantly enhances their microwave absorption performance, particularly in the K_u_ band. Specifically, the optimized GNS/CNF hybrids, when mixed with paraffin at a 30 wt% ratio, exhibit a maximum microwave reflection loss of −44.1 dB at 14.6 GHz with a thickness of 1.5 mm. Their effective absorption bandwidth, defined as the frequency range with a reflection loss below −10 dB, spans the 12.5–17.4 GHz range, covering more than 80% of the K_u_ band. These results indicate that the GNS/CNF hybrids prepared via intercalation CVD are promising candidates for microwave absorption materials.

## 1. Introduction

The widespread application of microwave technology has led to severe electromagnetic pollution [1,2]. Hybrids constructed of 2D nanomaterials, such as transition metal dichalcogenides [3], MoS_2_ nanosheets [4,5,6,7], and graphene [8], have gained considerable attention for their lightweight and highly efficient electromagnetic wave absorption properties. Among these, graphene, which is derived from layered natural graphite, stands out due to its exceptional performance and widespread research interest. By intercalating natural graphite, a typical layered material with interlayers bonded by van der Waals forces, a series of graphite intercalation compounds (GICs) were prepared, exhibiting distinctive electrical and magnetic properties [9]. Building on the concept of graphite intercalation, intercalation polymerization technology has been further developed in recent years [10]. A series of monomers, such as aniline, pyrrole, and caprolactam, are intercalated into graphite interlayers, where they undergo in situ polymerization, leading to the exfoliation of graphite layers and the formation of graphene–polymer hybrids [11,12,13]. Precise control of the intercalation polymerization process allows for the modulation of the hybrid structure and interactions between graphene and the polymer, thereby enabling efficient tuning of microwave absorption performance [13]. Notably, recent findings suggest that during the intercalation polymerization of aniline, metal ions can be simultaneously introduced into the graphite interlayers and effectively immobilized by the resulting polymer molecules [14]. This is fascinating, as it indicates that the composition and structure of metal alloys or compounds confined within the two-dimensional restricted environment of graphite interlayers can be precisely tuned through the annealing process. This grants such materials highly tunable dielectric and magnetic parameters, enabling precise control over the impedance matching between the material and free space, a factor that significantly impacts their microwave absorption performance.

In this work, FeNi_3_ nanoparticles were synthesized in situ within graphite layers through co-intercalation polymerization followed by annealing. These nanoparticles were then employed as catalysts to grow carbon nanofibers via intercalation chemical vapor deposition (CVD). The progressively growing carbon nanofibers induce the exfoliation of highly conductive graphite sheets and intertwine to create a porous structure, resulting in a sudden shift in the material’s electrical properties. This, in turn, impacts its impedance matching and effectively regulates its microwave absorption performance.

## 2. Materials and Methods

*Materials.* Natural graphite (100 mesh) was purchased from Nanjing Xianfeng nano-material technology Co., Ltd. (Nanjing, China). Aniline, ammonium peroxydisulfate, FeSO_4_·7H_2_O, NiSO_4_·6H_2_O, and concentrated sulfuric acid (98 wt%) used in the experiments were of analytical grade and were supplied by ALADDIN. It is worth noting that aniline was distilled under reduced pressure (35 mmHg, 85 °C) prior to use.

*Preparation of graphite nanosheets/carbon nanofiber (GNS/CNF) hybrids.* FeNi_3_ intercalated graphite was firstly prepared via our previously reported co-intercalation polymerization method [14]. Specifically, 0.2 g of natural graphite was dispersed in a solution prepared by dissolving 1 g of ammonium persulfate in 10 mL of concentrated sulfuric acid. The mixture was stirred continuously for 12 h, followed by centrifugation to separate the product. The obtained precipitate was then introduced into an aniline cation solution containing dissolved transition metal salts to initiate co-intercalation polymerization. This solution was prepared by dissolving 1.2 mL of aniline monomer, 13.1 g of FeSO_4_·7H_2_O, and 37.3 g of NiSO_4_·6H_2_O in 60 mL of 1 mol/L dilute sulfuric acid. The co-intercalation polymerization was carried out in an ice bath at 0–5 °C for 12 h. The resulting precipitate was then separated via vacuum filtration, repeatedly washed with ethanol and deionized water, and dried in an oven at 50 °C to obtain the Fe/Ni-polyaniline co-intercalated graphite compound. The as-prepared compounds were placed in a tube furnace for annealing for 30 min. The annealing temperature was set to 900 °C, with the atmospheric environment consisting of argon (Ar) and hydrogen (H_2_) gases. The gas flow rates were controlled at 100 sccm for argon and 5 sccm for hydrogen. Subsequently, the furnace was cooled to 650 °C for the CVD growth process. The atmospheric environment was adjusted to include argon (Ar), hydrogen (H_2_), and acetylene (C_2_H_2_) gases, with flow rates set to 40 sccm, 40 sccm, and 30 sccm, respectively.

*Characterization.* Electron micrographs were captured using a benchtop scanning electron microscope (EM-30^+^, coxemchina, Beijing, China) and a transmission electron microscope (TEM, Talos F200x, ThermoFisher Scientific, Waltham, MA, USA). The chemical structures of the samples were analyzed using Raman spectroscopy (LabRAM HR Evolution, employing a 532 nm laser, HORIBA, Ltd., Kyoto, Japan). Crystal structures were characterized using an X-ray diffractometer (D8 Advance, Bruker, Karlsruhe, Germany) with Cu Kα radiation. The electrical conductivity of the GNS/CNF hybrids was measured using a four-point probe instrument. Prior to testing, a fixed mass of the sample was pressed into a thin pellet under a pressure of 5 MPa. Electromagnetic parameters in the 2.0–18.0 GHz frequency range were measured using a vector network analyzer (E5071C, Agilent Technologies Inc., Santa Clara, CA, USA). To ensure the reproducibility of the test results, we prepared three sets of samples under each experimental condition and conducted tests on their electromagnetic parameters. For these tests, the powders were mixed with paraffin wax at a filler loading of 30 wt.%, then molded into toroidal-shaped samples with an outer diameter of 7.0 mm and an inner diameter of 3.04 mm.

## 3. Results

**Figure 1:**
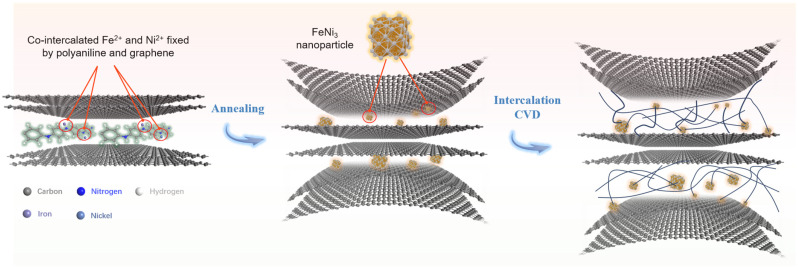
Schematic illustration of the mechanism for the preparation of graphite nanosheet/carbon nanofiber hybrids via the intercalation CVD technique.

The intercalation of metal ions into the graphite interlayer has been confirmed as a feasible process by co-intercalation polymerization of aniline [14]. This method enables the introduction of various metal ions into the graphite interlayer with precise control over their content and ratios, facilitating the formation of metal nanoparticles or metal alloy nanoparticles within the graphite interlayer. FeNi_3_ nanoparticles dispersed between graphite layers were used as catalysts to grow carbon nanofibers from the interlayer spaces of graphite via chemical vapor deposition, thereby exfoliating the graphite sheets and forming a hybrid structure composed of graphite nanosheets and carbon nanofibers. Figure 1 illustrates the technical process. Graphite intercalated with FeNi_3_ nanoparticles is denoted as FeNi_3_-NG, while the final products, consisting of graphite nanosheets and carbon nanofibers, are referred to as GNS/CNF hybrids.

**Figure 2:**
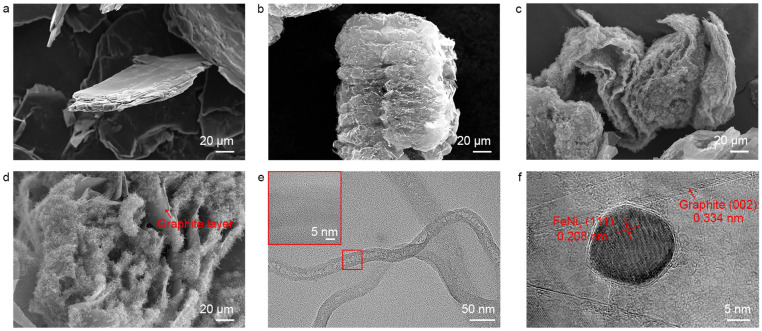
Electron microscopy characterizations of the samples. (**a**,**b**) SEM images of (**a**) natural graphite, (**b**) FeNi_3_-intercalated graphite. (**c**,**d**) Graphite nanosheet/carbon nanofiber hybrids prepared by intercalation CVD. (**e**) TEM images of the grown carbon nanofibers. The inset in the top left shows the high-resolution transmission electron microscopy (HR-TEM) image of the carbon fiber wall. (**f**) HRTEM images of a FeNi_3_ nanoparticle embedded between the graphite nanosheets. High-resolution lattice of FeNi_3_ (111) and graphite (002) planes are labeled.

The morphology of the samples during the intercalation CVD process was characterized, as shown in Figure 2. Natural graphite exhibits highly stacked layers with a smooth surface (Figure 2a). After co-intercalation polymerization with metal ions and aniline, followed by subsequent annealing at 900 °C for 30 min in a mixed atmosphere of argon and hydrogen, the graphite layers expand to form an expanded graphite-like morphology (Figure 2b). The metal ions between the graphite layers aggregate into nanoparticles, appearing as bright spots in the SEM images. The high-resolution TEM (HRTEM) characterization of the nanoparticles is shown in Figure 2c. Nanoparticles with a diameter of approximately 15 nm are embedded between the graphite nanosheets. Lattice calibration confirms that these are FeNi_3_ nanoparticles, which is consistent with reports in the existing literature [14]. Using the as-prepared FeNi_3_-NG as a catalyst, GNS/CNF hybrids were synthesized via CVD treatment at 650 °C in a mixed atmosphere of argon, hydrogen, and acetylene for 30 min. The SEM image of the resulting material is shown in Figure 2c,d. Abundant fibers emerge from the interlayers of graphite, leading to further separation of the graphite sheets. Figure 2e shows the TEM characterization results of a single fiber. The obtained fibers exhibit a hollow tubular structure. However, from the further magnified HR-TEM image, no graphitic lattice structure was observed. This confirms that the obtained fibers are not carbon nanotubes but rather hollow carbon fibers [15].

**Figure 3:**
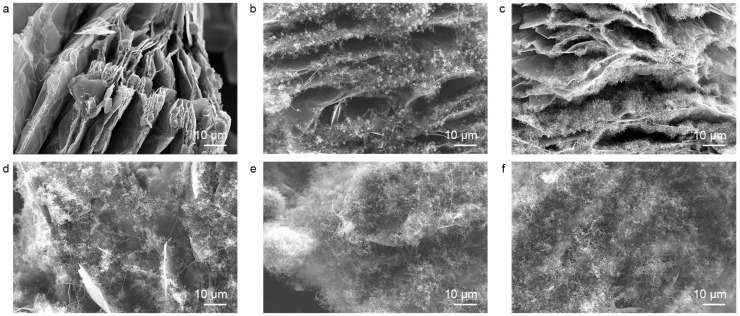
Scanning electron microscopy (SEM) images of graphite nanosheet/carbon nanofiber hybrids grown for different CVD durations. The specific CVD growth times are (**a**) 0 min, (**b**) 10 min, (**c**) 30 min, (**d**) 60 min, (**e**) 120 min, and (**f**) 240 min.

To analyze the structural evolution of GNS/CF hybrids during the intercalation CVD process, we controlled the duration of CVD growth after introducing the acetylene/argon gas mixture. The hybrid products obtained at different growth durations were characterized by SEM. Before introducing acetylene to initiate the CVD growth process, the co-intercalation polymerization-derived graphite transformed into an expanded graphite structure during the pre-annealing process (Figure 3a). A certain degree of separation appeared between the layers, but the structure remained predominantly stacked. When the CVD growth process proceeded for 10 and 30 min, clusters of fluffy fibers gradually grew on the graphite sheets. The fibers growing from between the graphite layers progressively pried apart the stacked graphite sheets, causing their separation (Figure 3b,c). When the growth continued to 60 min, the carbon fibers grew significantly longer and completely separated the graphite nanosheets, forming a hybrid structure composed of graphite nanosheets and carbon nanofibers (Figure 3d). In the subsequent growth process, the carbon fibers further elongated and completely encapsulated the graphite nanosheets, isolating them (Figure 3e,f).

**Figure 4:**
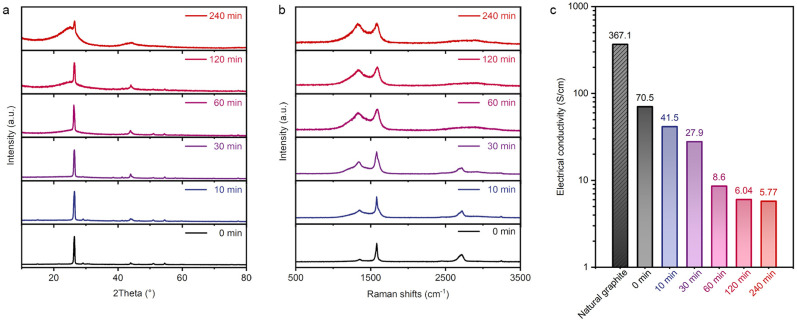
Structural characterizations and electrical conductivities of graphite nanosheet/carbon nanofiber hybrids grown for different CVD durations. (**a**) XRD patterns, (**b**) Raman spectra, and (**c**) electrical conductivities tested by four-point probe resistivity measurement.

We characterized and tested the structure and electrical conductivity of this series of products. Figure 4a demonstrates the XPD patterns of the samples. The sharp diffraction peak near 2θ = 26.4° corresponds to the (002) stacking diffraction peak of graphite. The diffraction peaks located at 2θ = 44° and 51° are caused by the lattice diffraction of FeNi_3_ [14]. With the increase in intercalation CVD growth time, especially after 60 min, a diffuse diffraction peak appeared near 2θ = 21°, while the relative intensity of the graphite (002) diffraction peak gradually decreased. The diffuse diffraction peaks, along with the TEM characterizations shown in Figure 2f, further confirmed that the fibers obtained from CVD growth were carbon nanofibers composed of amorphous carbon, rather than highly crystalline carbon nanotubes. Raman spectra in Figure 4b show the scattering signals of GNS/CNF hybrids. The peaks at 1353, 1583, and 2720 cm^−1^ correspond to the D band, G band, and 2D band of graphite [16]. The gradually increasing area ratio of the D peak to the G peak is likely caused by the growth of amorphous carbon fibers, and the broadened G peak and 2D peak are attributed to the exfoliation of graphite layers. It is well known that highly graphitized carbon nanomaterials, such as graphene and carbon nanotubes, exhibit high electrical conductivity, primarily due to the delocalized π-electron cloud and their well-ordered crystalline structure [17]. However, amorphous CNFs are often non-conductive, and ours are no exception. According to the four-probe conductivity measurements (Figure 4c), the conductivity of the natural graphite we used reached 367.1 S/cm. After co-intercalative polymerization of aniline with Fe/Ni metal ions and the pre-annealing treatment, the conductivity of the resulting products decreased to 70.5 S/cm. This is due to the initial expansion of graphite caused by annealing and the hybridization of FeNi_3_ that reduced the free electrons and decreased the carrier mobility. The growth of amorphous CNFs further caused a gradual decrease in the conductivity of the hybrids. When the CVD growth duration reached 60 min, the conductivity dropped sharply to 8.6 S/cm. Based on the morphology (Figure 3c,d) and structural characterization (Figure 4a,b), we believe this is due to the separation of graphite nanosheets caused by the growth of carbon fibers. Subsequent further growth of CNFs caused a slight decrease in conductivity to 5.77 S/cm, but the variation was not significant.

We used a vector network analyzer to measure the electromagnetic parameters of the samples to evaluate their microwave absorption performance. The GNS/CNF hybrids were mixed with molten paraffin and pressed into a coaxial ring before testing. The sample’s mass fraction in the mixture was 30 wt%. The electromagnetic parameters include complex permittivity (ε_r_ = ε′ − jε″) and complex permeability (μ_r_ = μ′ − jμ″). The real and imaginary parts of both the permittivity and permeability provide key insights into the material’s interaction with electromagnetic waves. The real part (ε′ or μ′) represents the material’s ability to store energy: ε′ describes how the material can store electric energy in response to an electric field, while μ′ indicates its ability to store magnetic energy in the presence of a magnetic field. The imaginary part (ε″ or μ″) corresponds to the energy dissipation ability of the material. Specifically, ε″ represents the loss of electric energy due to polarization processes, leading to heat generation, which is crucial for effective electromagnetic wave absorption. Similarly, μ″ reflects the magnetic energy dissipation, contributing to the material’s ability to absorb microwave energy. A higher imaginary part in either parameter indicates enhanced energy loss, making these materials suitable for applications in electromagnetic wave absorption.

**Figure 5:**
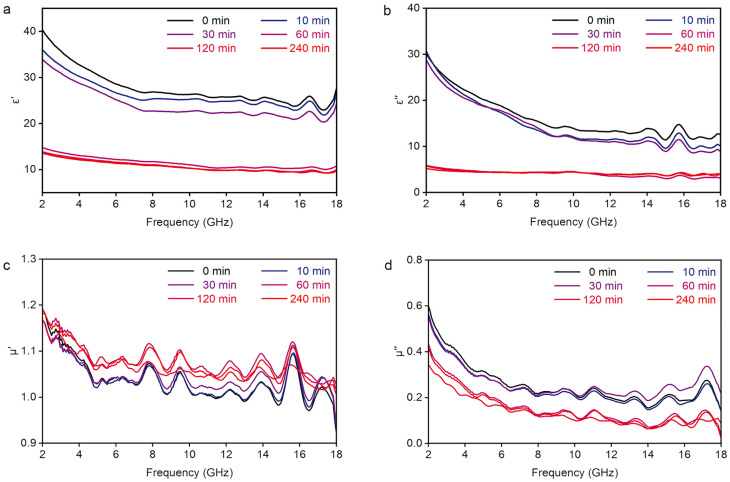
Frequency-dependent electromagnetic parameters of graphite nanosheet/carbon nanofiber hybrids grown for different CVD durations. (**a**) Real part and (**b**) imaginary part of the complex permittivity. (**c**) Real part and (**d**) imaginary part of the complex permeability.

For shorter CVD growth durations, less than 30 min, the growth of CNFs does not fully separate the graphite nanosheets, as discussed in Figure 3c and Figure 4. The resulting GNS/CNF hybrids exhibit relatively high values of ε′ and ε″. However, when the CNFs growth leads to complete separation of the graphite nanosheets, typically at a growth duration of 60 min, both ε′ and ε″ show a sharp decline (Figure 5a,b). In contrast, although the magnetic permeability (μ′ and μ″) also undergoes similar changes, the differences are not as pronounced (Figure 5c,d). The magnetism and magnetic loss in this material clearly stem from the ferromagnetic FeNi_3_, which acts as the catalyst. The variation in the relative permeability is likely associated with the FeNi_3_ content in the material. XRD patterns (Figure 4a) have confirmed that the catalyst composition does not undergo significant changes during the growth of CNFs. However, elemental analysis and ICP-MS measurements show a decrease in the relative proportions of Fe and Ni as the CVD growth time increases (Table S2), while the proportion of C increases accordingly. This is clearly attributed to the gradual growth of carbon fibers, particularly after 30 min.

**Figure 6:**
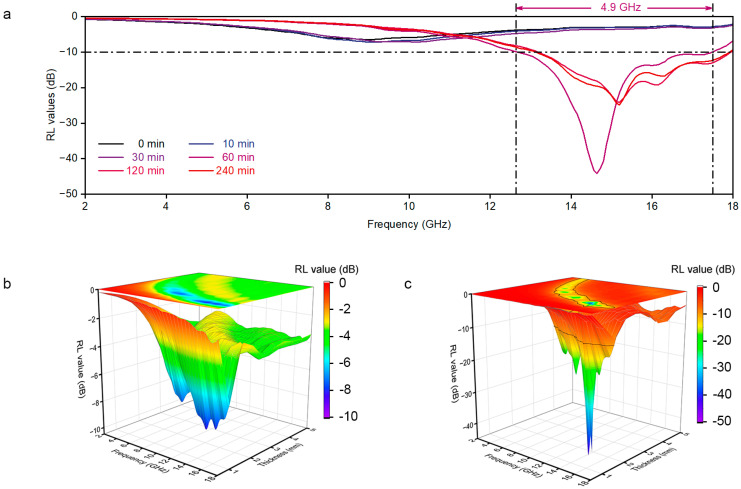
Microwave absorption performances of graphite nanosheet/carbon nanofiber hybrids grown for different CVD durations. (**a**) Reflection loss curves of the hybrids with a thickness of 1.5 mm. The black dashes indicate effective absorption regions (RL < −10 dB). (**b**,**c**) Three-dimensional representations of the hybrids grown for (**c**) 30 min and (**c**) 60 min. Note that the RL values are derived from the measured electromagnetic parameters.

We calculated the microwave reflection loss (RL) of the GNS/CNF hybrids using the measured electromagnetic parameters based on the transmission line theory. The specific calculation method is provided in Equation (1), where Z_in_ represents the input impedance of the absorbing material, and Z_0_ denotes the impedance of free space [14]. Figure 6a presents the RL curves of GNS/CNF hybrids prepared with different CVD growth durations as a function of microwave frequency, with an absorber thickness of 1.5 mm. To verify the reproducibility of the data, two additional sets of data are presented in Figures S1 and S2. For samples in which the graphite nanosheets were not exfoliated, specifically those with CVD growth durations of 0 min, 10 min, and 30 min, their reflection loss characteristics in the 2–18 GHz frequency range are similar, exhibiting very weak microwave absorption capabilities. However, once the graphite nanosheets are exfoliated by the grown CNFs, the reflection loss undergoes a dramatic change. The sample with a CVD growth duration of 60 min exhibits the most outstanding microwave absorption performance, with the strongest reflection loss reaching −44.1 dB at 14.6 GHz. Meanwhile, the effective absorption bandwidth, defined as the frequency range where the RL value is less than −10 dB, reaches 4.9 GHz, covering over 80% of the K_u_ band. As a comparison, we present the microwave absorption performance of carbon nanotube or carbon nanofiber/graphene hybrids reported in recent years, as shown in Table S1 [18,19,20,21,22,23,24,25]. Overall, the as-prepared GNS/CNF hybrids exhibit excellent and distinct microwave absorption properties. To gain a more comprehensive understanding of the differences in overall microwave absorption performance of the hybrids before and after the complete exfoliation of graphite nanosheets, we generated sets of 3D plots (Figure 6b,c and Figure S3). These plots illustrate the 3D surfaces of RL values as a function of microwave frequency and absorber thickness. The projections of the 3D surfaces are displayed at the top, with black lines outlining the effective absorption (RL < −10 dB) regions. It is evident that before the complete exfoliation of graphite nanosheets, the hybrids exhibit no effective absorption capability across any frequency or thickness within the evaluated range (Figure 6b). In contrast, the GNS/CNF hybrids obtained through the complete exfoliation of graphite nanosheets demonstrate excellent absorption performance within appropriate thickness and frequency ranges (Figure 6c). As for GNS/CNF hybrids prepared by further extending the CNF growth time to 120 min or even 240 min, the strongest absorption decreases slightly but still exhibits a relatively broad effective absorption capability within the Ku band (Figure 6a and Figure S3).

(1) $$RL=20\log\left|\frac{Z_{in}-Z_{0}}{Z_{in}+Z_{0}}\right|$$

**Figure 7:**
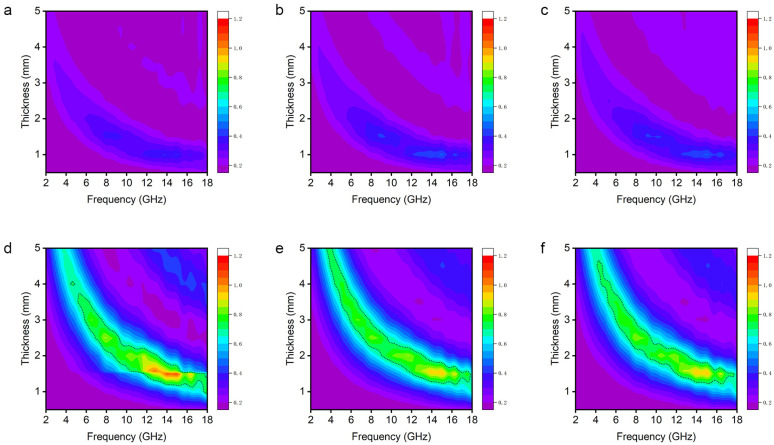
Impedance matches of graphite nanosheet/carbon nanofiber hybrids grown for different CVD durations. (**a**) 0 min, (**b**) 10 min, (**c**) 30 min, (**d**) 60 min, (**e**) 120 min, (**f**) 240 min.

Microwave absorption typically involves two processes. First, incident microwaves must penetrate into the interior of the material, a process determined by the impedance matching between the absorber and free space; subsequently, the microwaves that have entered the material should be converted into other forms of energy through loss mechanisms, thereby achieving microwave absorption loss [26,27,28]. Clearly, the impedance matching between the absorber and free space, which determines whether microwaves can pass through the absorber–air interfaces and enter the interior of the absorbing material, is a prerequisite for achieving microwave absorption. This parameter is typically measured using |Z_in_/Z_0_| and can be calculated using Equation (2) [29]. When this value approaches 1, it indicates that the input impedance of the absorber matches the impedance of free space more perfectly. Figure 7 illustrates the impedance matching calculation results for six groups of samples. Overall, before the exfoliation of the graphite nanosheets, the impedance matching of the hybrids is very poor (Figure 7a–c). This indicates that the majority of the incident microwaves are reflected at the air–absorber interface, preventing them from entering the interior of the absorber. From the morphological characterization results in Figure 3, it is observed that the conductive graphite nanosheets have not yet been completely exfoliated, resulting in the hybrids maintaining a relatively high conductivity (70.5 to 27.9 S/cm), as shown in Figure 4c. Generally, high conductivity tends to induce a strong electromagnetic shielding effect, causing the incident electromagnetic waves to be reflected rather than penetrating into the material [30].

(2) $$\left|\frac{z_{\mathrm{in}}}{z_{0}}\right|=\sqrt{\frac{\mu_{r}}{\varepsilon_{r}}}\tanh\;\left[j\left(\frac{2\pi fd}{c}\sqrt{\varepsilon_{r}\mu_{r}}\right)\right]$$

Here, μ_r_ and ε_r_ represent the complex permeability and permittivity, respectively, while d, *f*, and *c* denote the absorber thickness, incident microwave frequency, and the speed of light, respectively.

After the conductive graphite nanosheets are isolated from each other by the non-conductive CNFs, the abrupt change in electromagnetic parameters leads to a significant variation in impedance matching (Figure 7d–f). A crucial factor is the reduction in conductivity to 8.6–5.77 S/cm (Figure 4c), which significantly weakens the shielding effect. Another possible effect is that the growth of CNFs and their coating on GNSs create numerous pores within the nanostructure, which often facilitates further improvement in impedance matching.

**Figure 8:**
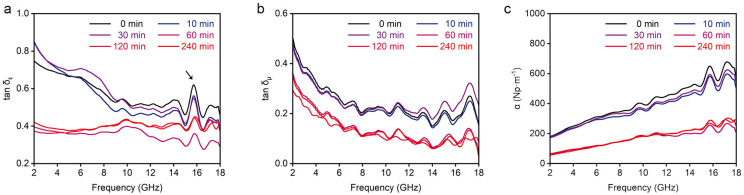
Microwave dissipation abilities of graphite nanosheet/carbon nanofiber hybrids. (**a**) Dielectric dissipation factors, (**b**) magnetic dissipation factor, and (**c**) attenuation value as a function of microwave frequency.

To analyze how the microwaves entering the absorber are dissipated and absorbed, we calculated the dielectric dissipation factor (tan δ_ε_) and magnetic dissipation factor (tan δ_μ_) based on the electromagnetic parameters. Their calculation formulas are given by Equations (3) and (4) [31]. A very obvious fact is that when the growth of CNFs separates and coats the graphite nanosheets, both the dielectric loss capacity and the magnetic loss capacity of the GNS/CNF hybrids decrease. All GNS/CNF hybrids exhibit a strong dielectric loss peak in the 16 GHz region as marked by the arrow in Figure 8a, which is attributed to the dielectric loss caused by the polarization relaxation of FeNi_3_ nanoparticles [14]. The dielectric loss exhibits particularly significant differences in the low-frequency region before and after the separation of graphite nanosheets. From the Cole–Cole curves (Figure S4), the dielectric loss in the low-frequency region is primarily in the form of conductive loss. Thus, it is easy to understand that the GNS/CNF hybrids with high electrical conductivity exhibit strong dielectric loss capability in the low-frequency region.

(3) $$\tan\delta_{E}=\frac{\varepsilon^{\prime \prime }}{\varepsilon^{\prime }}$$

(4) $$\tan\delta_{M}=\frac{\mu^{\prime \prime }}{\mu^{\prime }}$$

The magnetic loss similarly exhibits noticeable differences in the GNS/CNF hybrids before and after the separation of graphite nanosheets (Figure 8b). In fact, the magnetic properties of the CNS/CNF hybrids are almost entirely attributed to the magnetic FeNi_3_ [31,32]. And the major magnetic loss peak observed around 17 GHz has previously been confirmed to result from the loss induced by ferromagnetic resonance of FeNi_3_ nanoparticles intercalated between graphite layers [14]. Although the magnetic loss capability of the GNS/CNF hybrids is not particularly strong, it further diminishes after the graphite nanosheets are fully exfoliated and coated by CNFs. This reduction may be due to the extensive growth of carbon nanofibers, which encapsulate the FeNi_3_ nanoparticles serving as catalysts, thereby suppressing their magnetic characteristics. To assess the overall microwave loss performance of the CNS/CNF hybrids, the attenuation factor α was calculated using Equation (5) [33]. The variation of α with microwave frequency is presented in Figure 8c. It is clear that the overall loss capability is significantly reduced after the graphite nanosheets are exfoliated and coated with CNFs. Nevertheless, the final microwave absorption performance is remarkably enhanced. This improvement is evidently attributed to the significantly improved impedance matching, which plays a crucial role. The CNFs grown between graphite layers through CVD catalytic synthesis effectively separate and coat the highly conductive graphite sheets, while their entangled structure forms a porous network. This significantly enhances the impedance matching between the hybrids and free space, allowing more incident microwaves to penetrate into the absorber. Although the growth of CNFs reduces both dielectric and magnetic loss capabilities, proper structural optimization can strike a balance between impedance matching and loss performance, thereby achieving excellent microwave absorption properties.

(5) $$\alpha =\frac{\sqrt{2}\pi f}{c}\sqrt{\left(\mu^{\prime \prime }\varepsilon^{\prime \prime }-\mu^{\prime }\varepsilon^{\prime }\right)+\sqrt{{\left(\mu^{\prime \prime }\varepsilon^{\prime \prime }-\mu^{\prime }\varepsilon^{\prime }\right)}^{2}+{\left(\mu^{\prime \prime }\varepsilon^{\prime }+\mu^{\prime }\varepsilon^{\prime \prime }\right)}^{2}}}$$

## 4. Conclusions

Intercalation CVD is utilized to synthesize GNS/CNF hybrids with outstanding microwave absorption properties. FeNi_3_ nanoparticles, synthesized in situ within the graphite layers, act as catalysts to facilitate the growth of non-conductive CNFs from graphite interlayers. The highly conductive graphite sheets are progressively exfoliated and fully encapsulated by CNFs, resulting in the formation of GNS/CNF hybrids. During this process, the exfoliation of graphite sheets triggers a significant shift in the physical properties of the hybrids, resulting in abrupt changes in both conductivity and electromagnetic parameters. Although the microwave loss capability of the hybrids decreases during this process, the reduction in conductivity and the improved impedance matching with free space enable more incident microwaves to penetrate the absorbers. We believe that the porous structure resulting from the intertwining of carbon nanofibers plays a crucial role in enhancing impedance matching. As a result, by optimizing the material structure, exceptional microwave absorption performance in the K_u_ band can be achieved with an impressively thin thickness of only 1.5 mm. This study presents an interesting approach to the design and performance optimization of carbon-based microwave absorption materials, potentially offering valuable inspiration for researchers in this field.

## Data Availability

The original contributions presented in this study are included in the article/Supplementary Materials, further inquiries can be directed to the corresponding author/s.

## Supplementary Materials

The following supporting information can be downloaded at: https://www.mdpi.com/article/10.3390/nano15050406/s1, Figures S1 and S2: The microwave absorption performance of the microwave absorbing properties of two additional sets of parallel samples. Figure S3: Three-dimensional representations of the GNS/CNF hybrids grown for different CVD durations. Figure S4: Cole–Cole curves of the GNS/CNF hybrids grown for different CVD durations. Table S1: Comprehensive comparisons of MA performances for carbon fiber/nanotube and graphene hybrids reported recently. Table S2: The elemental percentages of each sample were calculated based on elemental analysis and ICP-MS.
