# Supplementary material for: Microwave Absorption Properties of Graphite Nanosheet/Carbon Nanofiber Hybrids Prepared by Intercalation Chemical Vapor Deposition
Source: Nanomaterials (Basel). 2025 Mar 6;15(5):406. doi: 10.3390/nano15050406 (PMC11901750; doi:10.3390/nano15050406)
Supplement: Supplementary file 1 [file nanomaterials-15-00406-s001.zip › nanomaterials-3427277-supplementary.pdf]

Supplementary

Microwave Absorption Properties of Graphite Nanosheet/Carbon Nanofiber Hybrids Prepared by Intercalation Chemical Vapor Deposition

Yifan Guo [1,*], Junhua Su [2], Qingfeng Guo [1], Ling Long [1], Jinlong Xie [3] and Ying Li [4]

[1] School of Aeronautical Equipment Manufacturing Industry, Chengdu Aeronautic Polytechnic, Chengdu 610100, China; guoqingfeng629@foxmail.com (Q.G.); sclongling@163.com (L.L.)

[2] Key Laboratory of Optoelectronic Technology & Systems (Ministry of Education), Center for Intelligent Sensing Technology, College of Optoelectronic Engineering, Chongqing University, Chongqing 400044, China; 20230801051g@stu.cqu.edu.cn

[3] School of Electronic Science and Engineering, University of Electronic Science and Technology of China, Chengdu 611731, China; 202111022814@std.uestc.edu.cn

[4] School of Mechanical Engineering, Chengdu University, Chengdu 610106, China; liying@cdu.edu.cn

* Correspondence: yfguo@cap.edu.cn

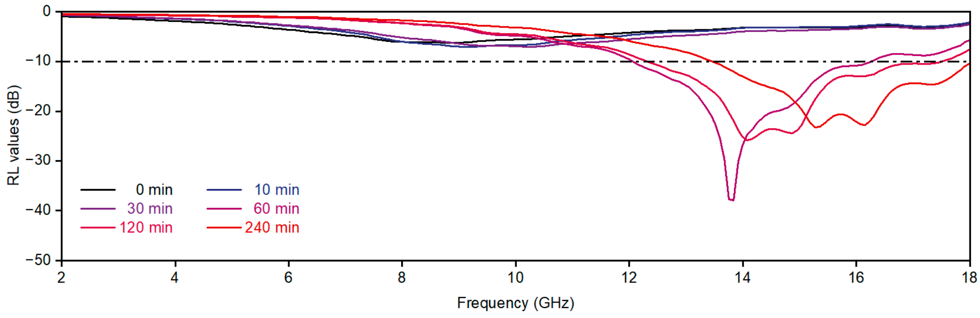

Figure S1. The microwave absorption performance of the first set of parallel samples. Note that the reflection loss curves of the hybrids are measured with a thickness of 1.5 mm.

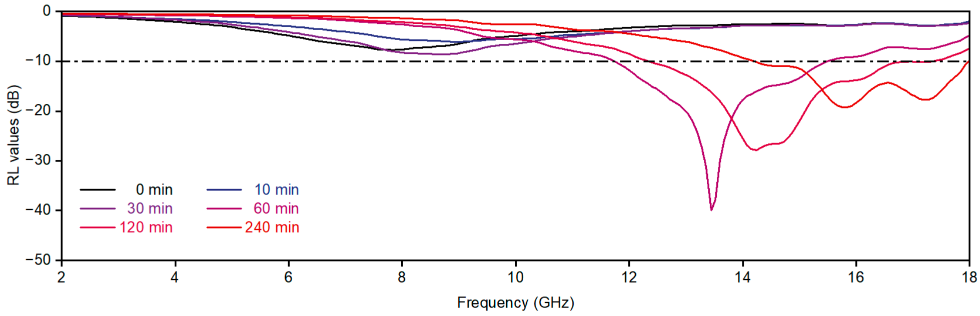

Figure S2. The microwave absorption performance of the other set of parallel samples. Note that the reflection loss curves of the hybrids are measured with a thickness of 1.5 mm.

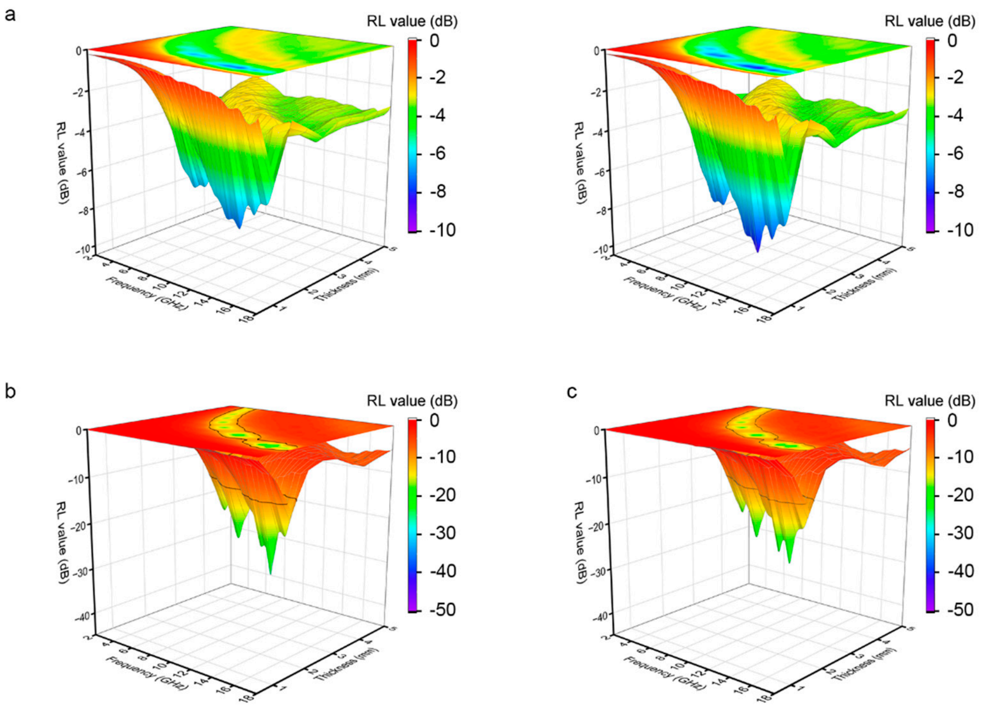

Figure S3. Three-dimensional representations of the GNS/CNF hybrids grown for different CVD durations. (a) 0 min, (b) 10 min, (c) 120 min, (d) 240 min.

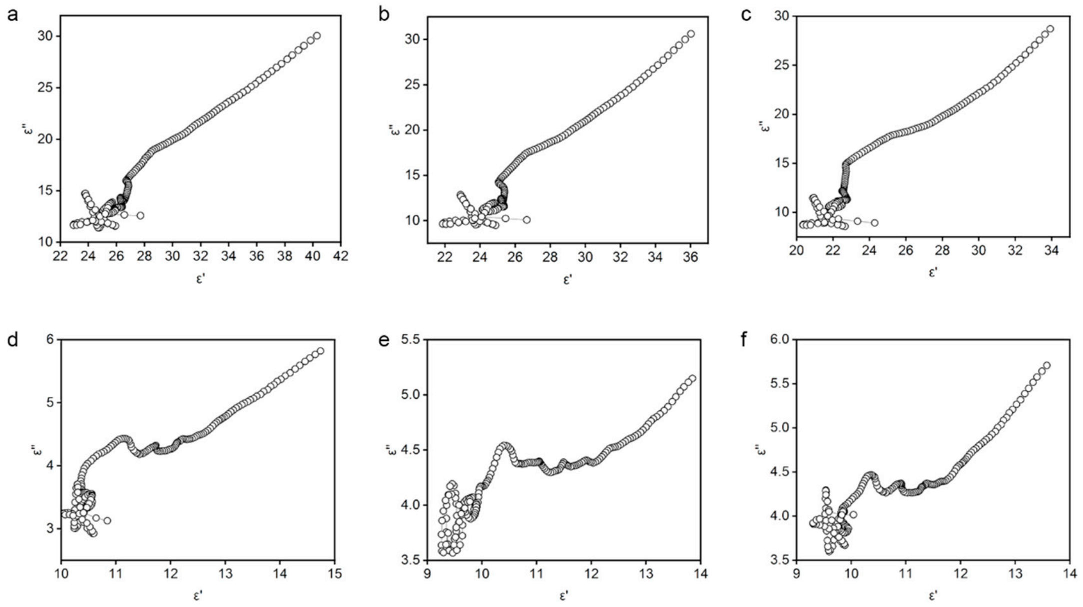

Figure S4. Cole-Cole curves of the GNS/CNF hybrids grown for different CVD durations. (a) 0 min, (b) 10 min, (c) 30 min, (d) 60 min, (e) 120 min and (f) 240 min.

Table S1 Comprehensive comparisons of MA performances for carbon fiber/nanotube and graphene hybrids reported recently.

Material	Matrix	Filler contents (wt%)	Thickness (mm)	Minimum RL value (dB)	Effective bandwidth (GHz)	References
GNS/CNF hybrids	Paraffin wax	30	1.5	-44.1	4.9	This work
CNTs/graphene	polyimide foam	—	3.3	-32.5	8.5	[18]
Carbon@graphene	Paraffin wax	20	1.5	-30.53	4.1	[19]
CNT-Fe_3O_4-graphene	Epoxy	—	0.25	-44.7	4.7	[20]
GO/CNT–Fe_3O_4	Paraffin wax	30	5	-37.25	0.89	[21]
CNTs/CoNi/graphene aerogels	Polydimethylsiloxane matrix	4	1.8	-50.8	8.5 (18-26.5 GHz)	[22]
CNT/graphene	Paraffin wax	—	10	-39.5	16	[23]
CNT/carbon	Paraffin wax	7	3	-43.6	7.42	[24]
CNTs@Ni/C	Paraffin wax	66.3	1.8	-47.0	5.8	[25]

References

[18] Y.-Y. Wang, W.-J. Sun, K. Dai, D.-X. Yan, Z.-M. Li, Flexible and heat-resistant carbon nanotube/graphene/polyimide foam for broadband microwave absorption, Composites Science and Technology 212 (2021) 108848.

[19] C. Wang, Y. Ding, Y. Yuan, X. He, S. Wu, S. Hu, M. Zou, W. Zhao, L. Yang, A. Cao, Y. Li, Graphene aerogel composites derived from recycled cigarette filters for electromagnetic wave absorption, Journal of Materials Chemistry C 3(45) (2015) 11893-11901.

[20] J. Li, W. Lu, J. Suhr, H. Chen, J.Q. Xiao, T.-W. Chou, Superb electromagnetic wave-absorbing composites based on large-scale graphene and carbon nanotube films, Scientific Reports 7(1) (2017) 2349.

[23] L. Wang, X. Jia, Y. Li, F. Yang, L. Zhang, L. Liu, X. Ren, H. Yang, Synthesis and microwave absorption property of flexible magnetic film based on graphene oxide/carbon nanotubes and Fe3O4 nanoparticles, Journal of Materials Chemistry A 2(36) (2014) 14940-14946.

[22] B. Zhao, Y. Li, H. Ji, P. Bai, S. Wang, B. Fan, X. Guo, R. Zhang, Lightweight graphene aerogels by decoration of 1D CoNi chains and CNTs to achieve ultra-wide microwave absorption, Carbon 176 (2021) 411-420.

[23] H. Chen, Z. Huang, Y. Huang, Y. Zhang, Z. Ge, B. Qin, Z. Liu, Q. Shi, P. Xiao, Y. Yang, T. Zhang, Y. Chen, Synergistically assembled MWCNT/graphene foam with highly efficient microwave absorption in both C and X bands, Carbon 124 (2017) 506-514.

[24] Y.-Y. Wang, Z.-H. Zhou, J.-L. Zhu, W.-J. Sun, D.-X. Yan, K. Dai, Z.-M. Li, Low-temperature

carbonized carbon nanotube/cellulose aerogel for efficient microwave absorption, Composites Part B: Engineering 220 (2021) 108985.
[25] D. Liu, L. Yang, F. Wang, H. Zhang, J. Liu, T. Lv, H. Zhao, Y. Du, Hierarchical carbon nanotubes@Ni/C foams for high-performance microwave absorption, Carbon 196 (2022) 867-876.

Table S2. The elemental percentages of each sample were calculated based on elemental analysis and ICP-MS.

Samples (CVD growth time)	Carbon content (at.%)	Iron content (at.%)	Nickel content (at.%)	Other element (at.%)
0 min	91.9	0.76	2.60	4.74
10 min	92.1	1.18	4.17	2.55
30 min	93.8	0.89	3.17	2.14
60 min	97.0	0.30	0.91	1.79
120 min	97.8	0.12	0.34	1.74
240 min	97.9	0.14	0.37	1.59
